# Reactions of Recombinant Neuronal Nitric Oxide Synthase with Redox Cycling Xenobiotics: A Mechanistic Study

**DOI:** 10.3390/ijms23020980

**Published:** 2022-01-17

**Authors:** Mindaugas Lesanavičius, Jean-Luc Boucher, Narimantas Čėnas

**Affiliations:** 1 Department of Xenobiotics Biochemistry, Institute of Biochemistry of Vilnius University, Saulėtekio 7, LT-10257 Vilnius, Lithuania; mindaugas.lesanavicius@gmc.vu.lt; 2 Laboratoire de Chimie & Biochimie Pharmacologiques et Toxicologiques, CNRS UMR 8601, Université Paris Descartes, 45 rue de Saints Pères, CEDEX 06, 75270 Paris, France; jean-luc.boucher@parisdescartes.fr

**Keywords:** nitric oxide synthase, quinones, nitroaromatic compounds, aromatic *N*-oxides, reduction mechanism, oxidative stress

## Abstract

Neuronal nitric oxide synthase (nNOS) catalyzes single-electron reduction of quinones (Q), nitroaromatic compounds (ArNO_2_) and aromatic *N*-oxides (ArN → O), and is partly responsible for their oxidative stress-type cytotoxicity. In order to expand a limited knowledge on the enzymatic mechanisms of these processes, we aimed to disclose the specific features of nNOS in the reduction of such xenobiotics. In the absence or presence of calmodulin (CAM), the reactivity of Q and ArN → O increases with their single-electron reduction midpoint potential (*E*^1^_7_). ArNO_2_ form a series with lower reactivity. The calculations according to an “outer-sphere” electron transfer model show that the binding of CAM decreases the electron transfer distance from FMNH_2_ to quinone by 1–2 Å. The effects of ionic strength point to the interaction of oxidants with a negatively charged protein domain close to FMN, and to an increase in accessibility of the active center induced by high ionic strength. The multiple turnover experiments of nNOS show that, in parallel with reduced FAD-FMN, duroquinone reoxidizes the reduced heme, in particular its Fe^2+^-NO form. This finding may help to design the heme-targeted bioreductively activated agents and contribute to the understanding of the role of P-450-type heme proteins in the bioreduction of quinones and other prooxidant xenobiotics.

## 1. Introduction

**Scheme 1:**
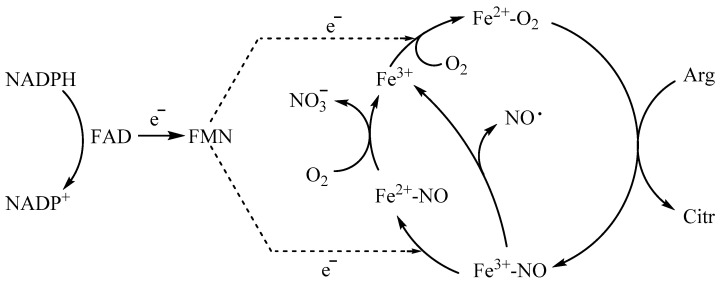
A simplified scheme of catalysis of nNOS (adapted from [2,13,14]).

Nitric oxide (NO) is produced physiologically to perform a range of signaling functions and as an immune response agent [1]. The mammalian NO synthases (NOS, EC 1.14.13.39) are dimeric flavohemoproteins that catalyze the conversion of *L*-arginine to NO· and citrulline at the expense of NADPH. The monomer of NOS consists of a heme domain with a tetrahydrobiopterin (H_4_B) bound at its *N*-terminus, and a FAD- and FMN-containing reductase domain at its *C*-terminus. The reductase and oxygenase domains are linked by a calmodulin (CAM)-binding sequence ([2,3], and references therein). The FAD-FMN domain of NOS is highly similar to that of microsomal NADPH:cytochrome P-450 reductase (P-450R, EC 1.6.2.4) [4,5,6]. The redox equivalents are transferred along the pathway NADPH → FAD → FMN → heme (tetrahydrobiopterin), and the FAD-FMN domain of NOS cycles mainly between one- and three-electron reduced states in the turnover [7,8,9,10]. The multistep NO· synthesis by NOS involves the formation of several heme Fe^3+^/Fe^2+^ complexes (Scheme 1) and electron exchange between the heme and H_4_B [2,11]. Importantly, only part of the NO· synthesized by NOS is released from the enzyme, because a significant fraction of its Fe^2+^-NO complex participates in the futile redox cycling which is not accompanied by NO· dissociation (Scheme 1) [12,13,14]. The catalysis of NOS involves the movement of FMN-containing domain and is regulated in a complex way by the binding of CAM, NADP(H), and the interdomain electrostatic interaction ([6,7,8,9,15,16,17] and references therein).

Like other flavoenzymes dehydrogenases-electron transferases, NOS reduces anticancer quinones (Q) and aromatic *N*-oxides (ArN → O), and toxic or therapeutic nitroaromatic compounds (ArNO_2_) in a single-electron way [8,10,18,19,20,21,22]. The radicals of the above compounds undergo redox cycling which may contribute significantly to the oxidative stress-type cytotoxicity. In particular, the neuronal form of NOS (nNOS) is the key factor in doxorubicin-induced toxicity to neurons [23], and may be involved in the neurotoxic action of dinitrobenzenes [24]. Because nNOS is overexpressed in astrocytic tumors, it may be regarded as a potential target for anticancer agents [25]. In turn, the inducible form of NOS (iNOS) is partly responsible for the reductive activation of anticancer agents 2-nitroimidazole EF5 and benzotriazine-*N*-oxide SN30000 [26]. In this case, its contribution was lower but still comparable to that of P-450R. Additionally, the NOS-catalyzed redox cycling of Q and ArNO_2_ may inhibit the formation of NO· because of its rapid reaction with the forming superoxide [24,27].

In spite of their importance, there exists a limited information on the mechanisms of reactions of NOS with redox cycling xenobiotics. The kinetic studies of flavin reductase domain of nNOS [8,10] and the insensitivity of quinone- and nitroreductase activity of holo-nNOS to *N*^ω^-nitro-*L*-arginine (NO_2_-Arg) that binds at the heme moiety lead to the conclusion that nNOS reduces the above compounds via FMNH_2_ and, possibly, FADH_2_ without the involvement of the heme [19,20,26]. In the absence of CAM, i.e., under the impeded electron transfer between the enzyme redox cofactors [16,17], nNOS shows little specificity towards the structure of quinones and nitroaromatic compounds, because their reactivity increases with their single-electron reduction midpoint potential (*E*^1^_7_) [22].

Extending our previous studies [22], we examined the reactions of nNOS with a number of oxidants of different structure, redox potential and electrostatic charge, with an emphasis on the specific aspects of reaction activation and regulation by the binding of CAM, NADP^+^, and the ionic strength of solution. Apart from the more comprehensive characterization of the functions of FMN domain, our data point to a parallel involvement of the heme moiety, in particular its Fe^2+^-NO complex, in the reduction of xenobiotics.

## 2. Results

### 2.1. Steady-State Kinetics and Substrate Specificity of nNOS

Previously we found that in the absence of CAM, quinones and nitroaromatic compounds form separate series of oxidants whose reactivity (log *k*_cat_/*K*_m_, where *k*_cat_/*K*_m_ is bimolecular reaction rate constant) increases with their *E*^1^_7_ [22]. The reactions were analyzed using an “outer-sphere” electron-transfer model, which describes an electron transfer with weak electronic coupling between the reactants ([28], and references therein), and the absence of pronounced structure specificity. In the simplest form, the rate constants of single-electron transfer between reagents (*k*_12_) depends on the electron self-exchange rate constants of reagents (*k*_11_ and *k*_22_) and equilibrium constant of reaction (*K*) (log *K* = ∆*E*^1^ (V)/0.059, where ∆*E*^1^ is the difference in the standard single-electron transfer potentials of reactants):

*k*_12_ = (*k*_11_ × *k*_22_ × *K* × *f*)^1/2^ (1)

and

log *f* = (log *K*)^2^/4log (*k*_11_ × *k*_22_/*Z*^2^) (2)

where *Z* is a frequency factor (10^11^ M^−1^s^−1^). According to Equations (1) and (2), in the reaction of electron donor with a series of homologous electron acceptors (*k*_22_ = constant), *k*_12_ will exhibit parabolic (square) dependence on ∆*E*^1^ with a slope ∆log *k/*∆∆*E*^1^ = 8.45 V^−1^ at ∆*E*^1^ = ±0.15 V. At ∆*E*^1^ = 0, *k*_12_ = (*k*_11_ × *k*_22_)^1/2^. Thus, the lower reactivity of ArNO_2_ was attributed to their *k*_22_ ~ 10^6^ M^−1^s^−1^ [29], which is much lower than that of quinones, 10^8^ M^−1^s^−1^ [30].

In this study, we examined the reactions of nNOS with a number of oxidants possessing a broad range of *E*^1^_7_ values and different electrostatic charge, including the anticancer agents such as the derivatives of tirapazamine (1–6) and dinitrobenzamide CB-1954 (24), and toxic environment pollutants tetryl (19) and 2,4,6-trinitrotoluene (20) (Table 1) ([8,9,18,19,20,21,22], and references therein). Because the *K*_m_ for NADPH in nNOS-catalyzed reactions is in the micromolar range [31], the measurements were performed at a single saturating concentration of NADPH, 50 µM. The maximal reduction rate constants (reaction turnover numbers, *k*_cat_) of these electron acceptors, and their *k*_cat_/*K*_m_ values are given in Table 1.

**Figure 1:**
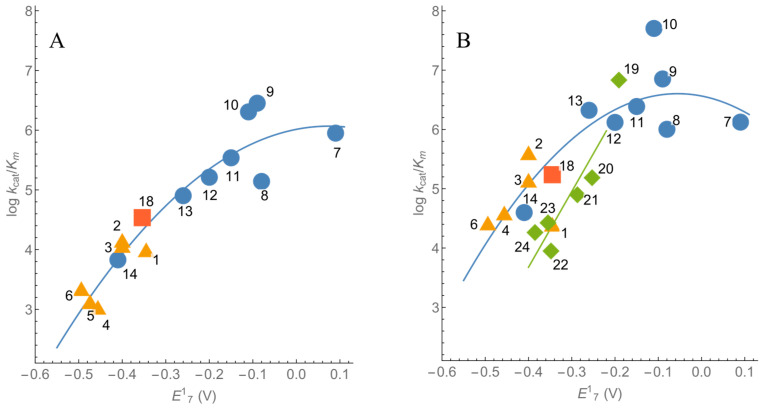
The dependence of the reactivity of quinones (blue disks), aromatic *N*-oxides (orange triangles), nitroaromatic compounds (green diamonds) and benzylviologen (red rectangle) on their single-electron reduction midpoint potentials (*E*^1^_7_) at pH 7.0 in the absence (**A**) and presence (**B**) of CAM and Ca^2+^. The numbers of compounds are given in Table 1.

First, we assessed the reactivity of a previously unexplored group of oxidants, ArN → O, in the absence of CAM. Figure 1A shows that log *k*_cat_/*K*_m_ of derivatives of 3-amino-1,2,4-benzotriazine-1,4-dioxide (tirapazamine), quinones and quinoidal single-electron acceptor benzylviologen, follow a common although scattered parabolic dependence on their *E*^1^_7_ values. According to an “outer-sphere” electron-transfer model [28], this may be attributed to their similar electron self-exchange rate constants, ~10^8^ M^−1^s^−1^ [30,34]. As expected, the addition of CAM significantly, but to a different extent, increased both the *k*_cat_ and *k*_cat_/*K*_m_ values of various groups of electron acceptors (Table 1). This resulted in a more scattered dependence of compound reactivity on their *E*^1^_7_ (Figure 1B). Nevertheless, the reactivity ArN → O remains similar to that of quinones. ArNO_2_ are less reactive than Q (Figure 1B), like in the experiments performed in the absence of CAM [22]. The addition of H_4_B decreased the rate constants of reduction of tetramethyl-1,4-benzoquinone (duroquinone) by 7–10%, and the addition of 1.0 mM dithiothreitol did not influence the reaction rate (data not shown). Importantly, the addition of 1.0 mM NO_2_-Arg decreased the *k*_cat_ of reaction by 25% (Table 1).

Next, we examined the effects of the ionic strength of the solution on the reactivity of electron acceptors. Electrostatic interactions play important role in the electron transfer reactions of FMN, first in the association of the FMN-binding domain with the FAD-binding domain, and, after the conformational change, in its complex formation with the heme domain [6,35,36,37,38,39]. However, most studies on the effects of ionic strength on the reactivity of nonphysiological oxidants were performed with their fixed concentrations, and provided limited information on the mode of their interaction with the FMN domain [35,37,38].

**Figure 2:**
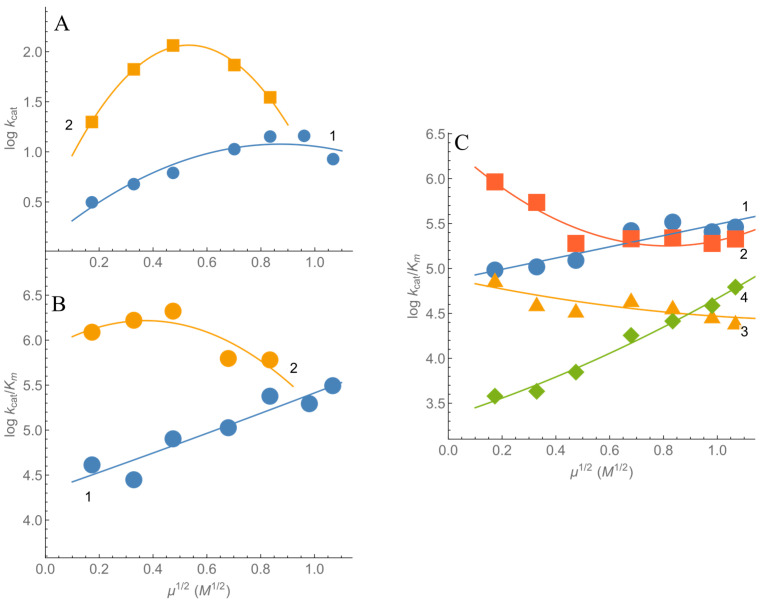
The effects of ionic strength on the reactivity of nNOS towards electron acceptors. (**A**) The dependence of *k*_cat_ of duroquinone on the ionic strength in the absence of calmodulin (1, blue disks) and its presence (2, orange squares). (**B**) The dependence of *k*_cat_/*K*_m_ of duroquinone on the ionic strength in the absence of calmodulin (1, blue disks), and its presence (2, orange disks). (**C**) The dependence of *k*_cat_/*K*_m_ for ferricyanide (1, blue disks), cytochrome *c* (2, red squares), Fe(EDTA)^−^ (3, green diamonds) and benzylviologen (4, orange triangles) on the ionic strength. [NADPH] = 50 μM, ionic strength varied by the addition of NaCl to 0.03 M Tris/HCl.

Using varied concentrations of uncharged oxidant duroquinone, we found that its *k*_cat_ attains the maximal value at high ionic strength (Figure 2A). Similar dependence is also characteristic for *k*_cat_ of cytochrome *c*, benzylviologen and ferricyanide (data not shown). On the other hand, *k*_cat_ of duroquinone attains a bell-shape dependence on the ionic strength in the presence of CAM (Figure 2A). The ionic strength also increases the *k*_cat_/*K*_m_ of duroquinone, but attenuates the activating effect of CAM (Figure 2B). The data of Figure 2C show that log *k*_cat_/*K*_m_ of negatively charged oxidants, ferricyanide and Fe(EDTA)^−^, increases with the ionic strength of solution. The decrease of the reactivity of positively charged cytochrome *c* and benzylviologen is less pronounced (Figure 2C). In general, these data point to the interaction of charged oxidants with the negatively charged FMN-binding domain (Figure 2C).

**Figure 3:**
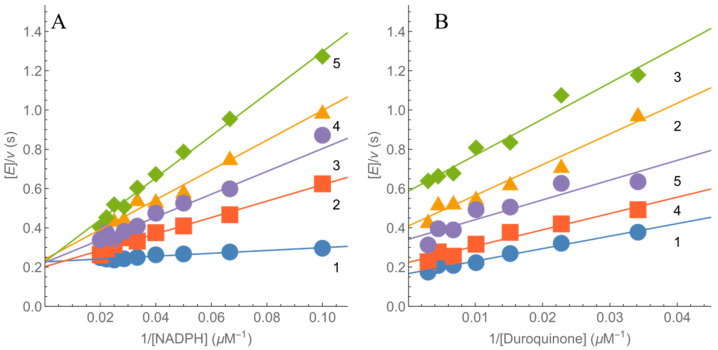
Inhibition of the duroquinone reductase reaction of nNOS by NADP^+^ and 2′,5′-ADP. (**A**) Inhibition at varied NADPH concentrations in the presence of 250 μM duroquinone: 1—inhibitor is absent, 2—200 μM NADP^+^, 3—400 μM NADP^+^, 4—50 μM 2′,5′-ADP, 5—100 μM 2′,5′-ADP. (**B**) Inhibition at varied concentrations of duroquinone in the presence of 50 μM NADPH: 1—inhibitor is absent, 2—1.0 mM NADP^+^, 3—2.0 mM NADP^+^, 4—250 μM 2′,5′-ADP, 5—500 μM 2′,5′-ADP. [E] is the enzyme concentration (M), and *v* is reaction rate (Ms^−1^).

Another insufficiently elucidated problem in the reduction of nonphysiological oxidants by nNOS, is the character of inhibition by reaction product, NADP^+^, and its relationship with NADP(H)-induced conformational transitions [15,31]. Figure 3A,B demonstrates typical inhibition patterns obtained at several concentrations of NADP^+^ and its redox inactive derivative 2′,5′-ADP. At a fixed concentration of duroquinone, both compounds act as competitive inhibitors to NADPH, because they decrease *k*_cat_/*K*_m_ of NADPH, but do not affect the *k*_cat_ value of the reaction (Figure 3A). This shows that they bind at the NADPH-binding site in the oxidized enzyme form. Figure 3B shows that at a fixed concentration of NADPH, both compounds act as noncompetitive inhibitors to duroquinone, decreasing its *k*_cat_/*K*_m_ and *k*_cat_ of the reaction.

The noncompetitive inhibition constants (*K*_i_) were calculated according to Equation (3) at 5–6 concentrations of NADP^+^ or 2′,5′-ADP (data not shown):

*v*/[E] = *k*_cat_ [Q]/(*K*_m_ + [Q]) × (1+ [I]/*K*_i_) (3)

where Q stands for oxidant and I stands for inhibitor. They were equal to 2.5 ± 0.2 mM for NADP^+^ and 0.25 ± 0.04 mM for 2′,5′-ADP. The decrease of *k*_cat(app.)_ in the presence of high concentrations of NADP^+^ and 2′,5′-ADP (Figure 3B) is most probably related to an increased *K*_m_ of NADPH (Figure 3A). In this case, 50 µM NADPH is no longer a saturating concentration and does not ensure the maximal reaction rate.

### 2.2. Kinetics of nNOS Oxidation under Multiple Turnover Conditions

**Figure 4:**
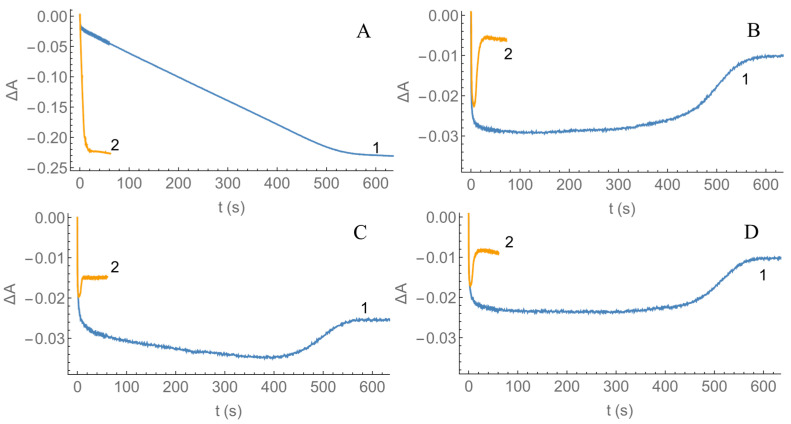
Multiple turnover kinetics of nNOS reduction and reoxidation and NADPH oxidation by O_2_ (1) or 100 µM duroquinone (2) in the absence of CAM. The absorbance changes were followed at 340 nm (**A**), 485 nm (**B**), 397 nm (**C**), and 436 nm (**D**). Concentrations of nNOS and NADPH after mixing, 2.0 µM and 30 µM.

In order to get insight into the mechanism of nNOS reoxidation by quinones, we investigated the changes of flavin and heme absorbance during enzyme multiple turnover under aerobic conditions. The reduction of flavins and heme was monitored following their absorbance decrease at 485 nm (heme isosbestic point) and 397 nm, respectively, whereas the formation of Fe^2+^-NO complex was monitored following the absorbance increase at 436 nm [13,14,40]. In parallel, the oxidation of NADPH was monitored according to the absorbance decrease at 340 nm. The data of Figure 4 show that nNOS was rapidly reduced by NADPH and slowly reoxidized by O_2_. Importantly, the amplitude of absorbance changes at 397 nm was comparable with those at 485 nm, pointing to a strong contribution of flavin spectral changes. The 436 nm absorbance changes are opposite to those expected for Fe^2+^-NO formation [13,40], and are attributed to FAD-FMN reduction. Following the oxidation by O_2_, the 485 nm, 397 nm and 436 nm absorbance does not return to an initial level, which indicates that the reduction product is stable single-electron reduced FAD-FMN [7,8,9,10,40]. The final drop of absorbance at 397 nm may be also partly caused by the oxidation of NADPH, which still weakly absorbs at this wavelength. Next, duroquinone, which does not possess absorbance in this region, was used as the electron acceptor. Its addition increases the enzyme reoxidation rate by more than one order of magnitude and decreases the amplitude of absorbance changes (Figure 4A–D).

**Figure 5:**
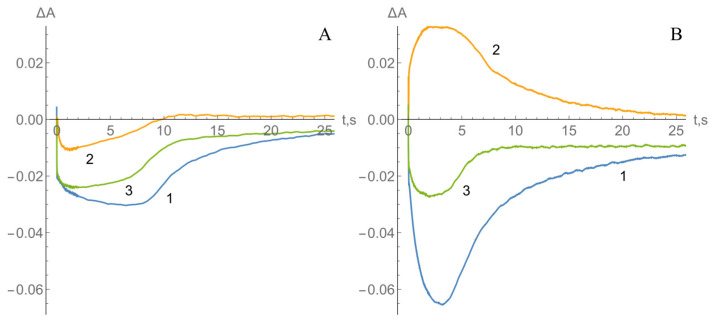
Multiple turnover kinetics of nNOS reduction and reoxidation by O_2_ in the presence of NADPH, CAM, H_4_B (**A**) and NADPH, CAM, H_4_B and Arg (**B**). The absorbance changes were followed at 397 nm (1), 436 nm (2). 485 nm (3). Concentrations of nNOS and NADPH after mixing, 2.0 µM and 30 µM.

The presence of CAM and H_4_B significantly enhanced the turnover of nNOS (Figure 5A). Like in the control experiments (Figure 4A–D), the addition of duroquinone accelerated the reoxidation of nNOS by one order of magnitude (data not shown). In order to study the full turnover of enzyme and to assess the roles of flavins and heme in the reduction of xenobiotics, all the subsequent experiments were performed in the presence of NOS substrate, arginine. Its addition to the reaction mixture led to an increase of absorbance at 436 nm, indicating the formation of Fe^2+^-NO complex (Figure 5B).

The analysis of initial absorbance changes of nNOS at 485 nm (Figure 5B) shows that NADPH reduces flavins biphasically, with *k*_red_ of 144.7 ± 1.5 s^−1^ and 3.4 ± 0.2 s^−1^ for the fast and slow phases with 70% and 30% amplitude, respectively. The heme absorbance changes at 397 nm are characterized by *k*_red_ of 67.6 ± 2.8 s^−1^ and 1.05 ± 0.1 s^−1^, respectively, with their amplitudes of 50%. The kinetics of Fe^2+^-NO formation at 436 nm followed after the initial 15 ms absorbance drop due to flavin reduction, are characterized by *k*_red_ of 35.3 ± 2.4 s^−1^ and 1.1 ± 0.1 s^−1^ with 62% and 38% amplitude, respectively. The obtained rate constants are comparable with the ones obtained during reduction of nNOS by excess NADPH under aerobic conditions [40], and with the data of further studies [3]. A second slow phase may be attributed to subsequent processes, e.g., the start of enzyme multiple turnover. The maximal absorbance change at 485 nm was close to that expected using the value of Δε_485_ = 12.4 mM^−1^cm^−1^ for the absorbance difference between the oxidized and three-electron-reduced FAD-FMN domain of nNOS [41], i.e., to almost complete formation of three-electron-reduced form. For the estimation of the maximal concentration of Fe^2+^-NO complex, the absorbance difference between Fe^2+^-NO and Fe^3+^ forms of flavin-free nNOS heme domain, Δε_436_ = 34.1 mM^−1^cm^−1^ [13], was corrected for the absorbance difference between oxidized and three-electron-reduced flavin reductase domain, Δε_436_ = 11.2 mM^−1^cm^−1^ [41]. Applying the obtained value, Δε_436_ = 22.9 mM^−1^cm^−1^, one may conclude that during the turnover, 67% of heme exist in Fe^2+^-NO form (Figure 5B), which is comparable with the previously reported value, 81% [14].

**Figure 6:**
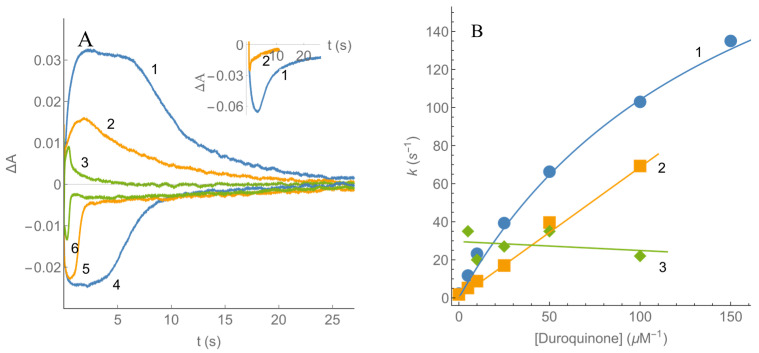
The kinetics of nNOS reduction and reoxidation under multiple turnover conditions in the presence of CAM, H_4_B and Arg. The concentrations of enzyme and compounds are given after mixing. (**A**) The spectral changes of 2.0 µM nNOS monitored at 436 nm (1–3) and at 485 nm (4–6) during the reduction by 30 µM NADPH and subsequent reoxidation by O_2_ (1, 4) and by 5.0 µM (2, 5) or 50 (3, 6) μM duroquinone. The inset shows the kinetics of spectral changes at 397 nm in the absence (1) and in the presence of 100 µM duroquinone (2). (**B**) Dependence of the apparent first order rate constants of reoxidation of reduced FAD-FMN (1), reoxidation of Fe^2+^-NO complex (2) and formation of Fe^2+^-NO complex (3) on duroquinone concentration.

The addition of duroquinone enhances the flavin reoxidation and decreases the amplitude of absorbance changes at 485 nm (Figure 6A). The kinetics of reoxidation were analyzed by the method of Chance [42] using Equation (4):

*k*_ox_ = [NADPH]_0_/([E_red_]_max_ × t_1/2(off)_) (4)

where *k*_ox_ is the apparent first-order rate constant of enzyme reoxidation, [NADPH]_0_ is the initial concentration of NADPH, [E_red_]_max_ is the maximal concentration of the reduced enzyme formed during the turnover, and t_1/2(off)_ is the time interval between the formation of the half-maximal amount of E_red_ and its decay to the half-maximal value.

The dependence of *k*_ox_ on duroquinone concentration (Figure 6B) corrected for *k*_ox_ for reaction with O_2_, 2.24 s^−1^, gives an apparent bimolecular rate constant of 1.7 ± 0.1 × 10^6^ M^−1^s^−1^, which is comparable with the steady-state *k*_cat_/*K*_m_ for this oxidant (Table 1), and *k*_ox(max)_ = 267 ± 30 s^−1^. Thus, using duroquinone as oxidant, the *k*_cat_ of the steady-state reaction is limited mainly by the reductive half-reaction. Although duroquinone accelerates nNOS oxidation followed at 397 nm with a similar rate (Figure 6A, inset), we further assessed the reoxidation of Fe^2+^-NO complex (Figure 6A), because this is the predominant heme form during the enzyme turnover [14], and its absorbance changes are opposite to those of flavin cofactors. One may note the analysis of this reaction according to Equation (4) is complicated by the branched catalytic cycle of nNOS with the parallel electron flux through several Fe^2+^ complexes (Scheme 1). After the conditional assumption that the maximal concentration of Fe^2+^-NO during turnover in the presence of O_2_ is equal to enzyme concentration and after the correction for *k*_ox_ of oxygen, 1.8 s^−1^, an apparent bimolecular rate constant of its reoxidation, 7.4 ± 0.85 × 10^5^ M^−1^s^−1^, is obtained (Figure 6B). The addition of 100 U/mL superoxide dismutase (SOD) did not affect the rate of Fe^2+^-NO reoxidation by duroquinone.

Analogously, the rate constants of Fe^2+^-NO formation during the enzyme reduction by NADPH (*k*_red_) were calculated according to Equation (5) [42], where *k*_ox_ is the rate constant of reoxidation at particular duroquinone concentration, and [E]_t_ is the total enzyme concentration:

*k*_red_ = *k*_ox_/([E]_t_/[E_red_]_max_ − 1) (5)

Although being scattered (Figure 6B), the obtained values of *k*_red_ were comparable with *k*_red_ = 35.3 s^−1^ obtained in the analysis of absorbance rise in the absence of duroquinone. Importantly, an increase in duroquinone concentration affects the value of *k*_red_ insignificantly (Figure 6B).

## 3. Discussion

Our study discloses several specific properties of nNOS relevant to the reduction of quinones, nitroaromatic compounds and aromatic *N*-oxides. First, there does not exist a well-defined oxidant specificity except for their reactivity increase with *E*^1^_7_, and the difference between two series of compounds, Q and ArN → O, and ArNO_2_ (Figure 1A,B). This shows that the model of an “outer-sphere” electron transfer ([28], and references therein) may be also applied for the reactions in the presence of CAM (Figure 1B), i.e., under close to physiological conditions. Importantly, the values of *k*_cat_/*K*_m_ of oxidants (Figure 1B) show that in the presence of CAM, nNOS becomes almost as active as rat P-450R [34]. This points to a possibility of their similar responsibility for the oxidative stress-type cytotoxicity of redox cycling xenobiotics in particular types of cells.

In this context, an important problem is the mechanism of the activation of reduction of external oxidants by CAM [7,8,37,38], which, to the best of our knowledge, is far less addressed than its effects on the intraprotein electron transfer [2,3,16,17]. In general, CAM accelerates the transitions between various conformational states of nNOS flavin reductase domain and favors the conformational states with the better FMN accessibility to solvent ([16,17], and references therein), however, it also possibly decreases the *E*^1^_7_ of FMNH·/FMNH_2_ couple and increases its reactivity [41]. Thus, it is of interest to assess the effect of CAM on the distance of electron transfer in the reduction of quinones by nNOS. According to an “outer-sphere” electron transfer model, the distances of electron transfer (*R*_p_) between metalloproteins and inorganic complexes may be obtained from the protein electron self-exchange rate constants (*k*_11_) [43]:

*R*_p_ (Å) = 6.3 − 0.35 ln *k*_11_ (6)

The application of Equation (6) for the reactions of quinones with single-electron transferring flavoenzymes gives *R*_p_ *=* 3.4–5.0 Å [44], which may be slightly overestimated because of the partial solvent accessibility of flavin cofactors. Thus, these values may be useful only for an approximate assessment of the “intrinsic” flavoenzyme reactivity. The *k*_11_ values of nNOS may be estimated from the data of Figure 1A,B at ∆*E*^1^_7_ = 0, where, according to Equation (1), the reaction rate constant (*k*_12_), is equal to (*k*_11_ × *k*_22_)^1/2^. In this case, the *E*^1^_7_ of the oxidant should be equal to that of FMNH·/FMNH_2_ couple. The results of calculations according to Equation (6) (Appendix A) show that in the presence of CAM, the estimated values of *R*_p_, 2.8–3.1 Å are lower than in its absence, 3.9–4.7 Å, i.e., a better access to FMN is apparently ensured. Therefore, in spite of a possible decrease of the redox potential of FMNH·/FMNH_2_ by CAM [40], the enhancement of nNOS reactivity is at least partly attributed to an increase in FMN accessibility. Importantly, in this case the *R*_p_ values of nNOS become similar to *R*_p_ for P-450R, 3.4 Å, calculated using the same method [44].

The studies of the ionic strength effects (Figure 2A–C) provide an additional information on the role of conformational changes of the FMN-binding domain in the reduction of external oxidants. The motions of the FMN-binding domain involve the interaction of negatively charged Glu762 with Arg1229 of the FAD domain, and, in an alternative conformation, the interaction of Glu762, Glu816 and Glu819 with Lys423, Lys620 and Lys660 of the heme domain [6,39]. On the other hand, high ionic strength enhanced the reduction of FAD by NADPH and the interflavin electron transfer, and increased flavin fluorescence intensity in CAM-free enzyme [38]. This partly mimicked the effects of CAM, and was interpreted as an induction of enzyme conformation with a better FMN accessibility to solvent. However, the data available so far do not allow to distinguish the role of electrostatic interactions of nNOS with the charged electron acceptors, and the possible effects of ionic strength on FMN accessibility. The use of *k*_cat_/*K*_m_ as a measure of reactivity enabled to demonstrate the existence of the ionic strength-induced conformational change which increases the reactivity of uncharged duroquinone (Figure 2B). Its contribution distorts the expected log *k*_cat_/*K*_m_ vs. µ^1/2^ dependence for the reactions of a negatively charged FMN-binding domain with the charged oxidants, as it is found in reactions of P-450R, where the limiting reactivity was attained at µ^1/2^ = 0.5–0.6 M^1/2^ [45]. Instead, the reactivity of negatively charged ferricyanide and Fe(EDTA)^−^ does not reach the limiting value even at µ^1/2^ = 1.0 M^1/2^ (Figure 2C), apparently because of a general increase in the FMN accessibility at high ionic strength.

Another problem related to the mechanism of reduction of external electron acceptors, is the possible modulation of the reaction rate by repeatedly bound NADP(H). The presteady-state studies of nNOS show that the binding of NADPH or NADP^+^ to the reduced flavin domain inhibits the reduction of external oxidants, evidently inducing a conformational change which restricts the accessibility of FMN [15]. The reaction is not inhibited by 2′,5′-ADP which lacks nicotinamide ring. However, our data (Figure 3A,B) and those of the previous study [31] do not demonstrate different character of inhibition by NADP^+^ and 2′,5′-ADP or 2′-AMP. In our case, NADP^+^ and redox inactive 2′,5′-ADP act as competitive inhibitors to NADPH (Figure 3A), competing for its binding site at the oxidized enzyme form. Their noncompetitive inhibition with respect to duroquinone, and the decrease of *k*_cat(app.)_ of reaction in particular (Figure 3B) is most probably caused by an increase of *K*_m_ of NADPH at high concentrations of NADP^+^ or 2′,5′-ADP (Figure 3A). In this case, 50 µM NADPH is no longer a saturating concentration and does not ensure the maximal reaction rate. Among other possible reasons, the discrepancy between the steady- and presteady-state data may be explained by the participation of four-electron reduced flavin domain of nNOS in the presteady-state experiments [15], whereas the steady-state turnover involves enzyme cycling between single- and three-electron reduced states [7,8,9,10].

The data of our study imply that using duroquinone as oxidant, the *k*_cat_ of the steady-state reaction is limited mainly by the reductive half-reaction (Figure 6B). Evidently, for most other oxidants, the *k*_cat_ is limited by the oxidative half-reaction (Table 1). The nature of this phenomenon deserves more thorough studies in the future. On the other hand, according to the previous studies, *N*^ω^-nitroarginine that binds at the heme moiety and prevents its reduction by FMNH_2_ [46], did not inhibit the reduction of nilutamide and CB-1954 by nNOS [19,20]. Thus, it was concluded that heme does not participate in the reduction of above compounds. However, the multiple turnover experiments show that, in parallel with the reduced FAD-FMN, duroquinone oxidizes the heme of nNOS, in particular its Fe^2+^-NO form (Figure 6A,B). In this context one may note that the previous studies [19,20] were performed in the absence of arginine, i.e., under the conditions where Fe^2+^-NO is not formed. The decrease of amplitude and lifetime of absorbance transient at 436 nm is not caused by the interception of electron flux from FMNH_2_ to heme, because the rate constant of Fe^2+^-NO formation, *k*_red_, insignificantly decreases with an increase in duroquinone concentration (Figure 6B). Another potential mechanism of a decay of Fe^2+^-NO is the dissociation of NO· driven by high rate of formation of peroxynitrite under the action of O_2_^−·^ formed during redox cycling of duroquinone [35]. However, the superoxide-driven decomposition of Fe^2+^-NO takes place only after addition of chaotropic agents that decrease the complex stability [35]. Moreover, our findings show that Fe^2+^-NO depletion by duroquinone is insensitive to SOD. On the other hand, Fe^2+^-NO is less reactive than reduced FAD-FMN (Figure 6B), and the rate of its formation, 35.3 s^−1^, is lower than the rate of reduction of FAD-FMN, 144.7 s^−1^, or the *k*_cat_ of steady-state reaction, 112.2 s^−1^ (Table 1). This shows that heme may provide only a minor part of electron flux for the reduction of external electron acceptors, and that FMNH_2_ remains a preferential electron donor. This is in line with an insignificant inhibition of reaction by NO_2_-Arg (Table 1). A similar example is flavohemoglobin, where during the turnover, quinones oxidize the heme much slower than the other redox center, reduced FAD [47]. In this case the azole compounds which bind at the heme moiety do not alter the *k*_cat_/*K*_m_ value for oxidant.

Currently, the involvement of heme moiety in reduction of drugs and xenobiotics by NOS is demonstrated only for iNOS-catalyzed reduction of the anticancer prodrug 1,4-bis(aminoalkyl-*N*-oxide)-anthracene-9,10-dione AQ4N, which is converted into its bis(aminoalkyl)-derivative [48]. The data of this study (Figure 6A,B) expand the variety of structures that may be activated in this way, thus opening a perspective for the design of heme-targeted bioreductively activated agents. The observation of a high reactivity of quinone towards the heme moiety of nNOS may also foster the more detailed studies in the related area, the cytochrome P-450-catalyzed single-electron reduction of quinones, aromatic *N*-oxides and nitrocompounds ([49,50,51], and references therein). Although these reactions are relevant to the cytotoxicity of the above compounds, their mechanisms are not disclosed at the molecular level because of the use of proteoliposomal preparations but not the isolated enzymes in the studies.

## 4. Materials and Methods

### 4.1. Enzymes and Reagents

Recombinant rat neuronal nitric oxide synthase (nNOS) was prepared as previously described [19]. Its concentration was determined spectrophotometrically according to ε_393_ = 100 mM^−1^cm^−1^ [52]. The activity of nNOS determined according to the assay of [^3^H]-*L*-citrulline formation [19], was equal to 280 ± 50 nmol × min^−1^ × mg protein^−1^. 2,4,6-Trinitrotoluene (TNT), 2,4,6-trinitrophenyl-*N*-methylnitramine (tetryl), 3-amino-1,2,4-benzotriazine-1,4-dioxide (tirapazamine) and its 7-substituted derivatives, synthesized as previously [22,33], were a generous gift of Dr. Jonas Šarlauskas (Institute of Biochemistry, Vilnius). 5-(1-Aziridinyl)-2,4-dinitrobenzamide (CB-1954), synthesized as described previously [53], was a generous gift of Dr. Vanda Miškinienė (Institute of Biochemistry, Vilnius). All the synthesized compounds were characterized by their melting points, ^1^H-NMR and IR spectra. Cytochrome *c*, NADPH, superoxide dismutase (SOD), calmodulin, *L*-arginine, *N*^ω^-nitro-*L*-arginine, tetrahydrobiopterin, dithiothreitol, benzylviologen, quinones and nitroaromatic compounds were obtained from Sigma-Aldrich (St. Louis, MO, USA) and used as received.

### 4.2. Steady-State Kinetic Studies

All kinetic experiments were carried out spectrophotometrically using a Cary 60 Agilent UV-Vis spectrophotometer (Santa Clara, CA, USA) in 0.1 M Tris-HCl buffer (pH 7.0) containing 0.1 M NaCl and 1.0 mM EDTA at 25 °C. In the experiments with varied ionic strength, different concentrations of NaCl were added to 0.03 M Tris-HCl (pH 7.0) The steady-state parameters of the reactions, namely the catalytic constants (*k*_cat_) and the bimolecular rate constants (or catalytic efficiency constants, *k*_cat_/*K*_m_) of the oxidants at fixed concentrations of NADPH (50 μM) were obtained by fitting the kinetic data to the parabolic expression using SigmaPlot 2000 (v. 11.0, SPSS Inc., Chicago, IL, USA). They correspond to the reciprocal intercepts and slopes of Lineweaver-Burk plots, [E]/*v* vs. 1/[oxidant], respectively, where *v* is the reaction rate and [E] is the enzyme concentration. *k*_cat_ represents the number of molecules of NADPH oxidized by a single active center of the enzyme per second under saturating conditions. The concentrations of nNOS used in the experiments were 10–50 nM. The rates of nNOS-catalyzed NADPH oxidation in the presence of quinones, nitroaromatic compounds or tirapazamine derivatives were determined using the value Δε_340_ = 6.2 mM^−1^cm^−1^. The rates were corrected for the intrinsic NADPH-oxidase activity of the enzyme, 0.1 s^−1^. The rates of reduction of cytochrome *c* and ferricyanide were determined using Δε_550_ = 20 mM^−1^cm^−1^ and Δε_420_ = 1.0 mM^−1^cm^−1^, respectively. In some experiments, the reaction medium was supplemented by 10 μg/mL calmodulin and 1.0 mM CaCl_2_, or by CAM-Ca^2+^ and H_4_B (5.0 µM), or by CAM-Ca^2+^ and H_4_B and *L*-arginine (200 µM). In this case, the rates were corrected for the intrinsic NADPH-oxidase activity of the enzyme, reaching 1.0–1.7 s^−1^.

### 4.3. Presteady-State Kinetic Studies

Enzyme rapid kinetic studies were performed using a SX20 stopped-flow spectrophotometer (Applied Photophysics, Leatherhead, UK) under aerobic conditions in 0.1 M Tris-HCl (pH 7.0) containing 0.1 M NaCl, 3% (*v*/*v*) glycerol at 25 °C. First syringe contained either nNOS (2.0 μM after mixing), or nNOS, CAM, CaCl_2_ and H_4_B (2.0 μM, 5.0 µM, 1.0 mM and 5.0 μM after mixing, respectively), while second syringe contained NADPH (30 μM after mixing), or NADPH and various concentrations of duroquinone, or NADPH and *L*-arginine (200 µM after mixing), or NADPH, *L*-arginine and duroquinone. In separate experiments, superoxide dismutase was added to a second syringe. All experiments were performed in triplicate. The kinetic traces at 485, 436 and 397 nm were analyzed using the accompanying software package by fitting it to single- or double-exponential equations. The multiple turnover data were analyzed by the method of Chance [42].

## 5. Conclusions

Our study shows that under particular conditions, the activity of nNOS in the bioactivation of prooxidant drugs and xenobiotics may be similar to that of P-450R, which is typically regarded as the most important enzyme responsible for their oxidative stress-type cytotoxicity. We characterized several specific properties of nNOS, in particular, the roles of accessibility of FMN and electrostatic interactions, in these reactions. In general, the model of an “outer-sphere” electron transfer which does not predict a pronounced oxidant structure specificity, may be applied for the analysis of currently available data and approximate prediction of reactivity of unexplored quinones, nitroaromatic compounds and aromatic *N*-oxides. However, the demonstration of an involvement of the heme moiety in the reduction of external oxidants may open new perspectives for the design of new specific heme-targeted structures of compounds.

## Figures and Tables

**Table 1:** Steady-state rate constants of the reduction of nonphysiological electron acceptors by NADPH catalyzed by nNOS. [NADPH] = 50 μM, 0.1 M Tris/HCl + 1 mM EDTA + 100 mM NaCl, pH 7.0, 25 °C; 10 μg/mL calmodulin (CAM) and 1.0 mM CaCl_2_, where applicable.

No.	Compound	*E*^1^_7_ (V) [32,33]	−CAM		+CAM	
			*k*_cat_ (s^−1^)	*k*_cat_/*K*_m_ (M^−1^s^−1^)	*k*_cat_ (s^−1^)	*k*_cat_/*K*_m_ (M^−1^s^−1^)
**Aromatic *N*-oxides**						
1.	7-CF_3_-tirapazamine	−0.345	3.98 ± 0.38	9.9 ± 1.2 × 10^3^	16.3 ± 1.8	2.5 ± 0.2 × 10^4^
2.	7-Cl-tirapazamine	−0.400	0.84 ± 0.08	1.4 ± 0.1 × 10^4^	14.8 ± 1.7	4.0 ± 0.6 × 10^5^
3.	7-F-tirapazamine	−0.400	2.84 ± 0.23	1.2 ± 0.1 × 10^4^	11.1 ± 1.1	1.4 ± 0.1 × 10^5^
4.	3-Amino-1,2,4-benzotriazine-1,4- dioxide (tirapazamine)	−0.456	0.69 ± 0.09	1.1 ± 0.1 × 10^3^	5.22 ± 0.66	3.9 ± 0.2 × 10^4^
5.	7-CH_3_-tirapazamine	−0.474	0.60 ± 0.09	1.3 ± 0.2 × 10^3^	ND	ND
6.	7-C_2_H_5_O-tirapazamine	−0.494	0.67 ± 0.09	2.2 ± 0.2 × 10^3^	2.94 ± 0.71	2.6 ± 0.2 × 10^4^
**Quinones**						
7.	1,4-Benzoquinone	0.090	12.9 ± 1.5	5.1 ± 0.7 × 10^5^	61.4 ± 7.2	1.3 ± 0.2 × 10^6^
8.	2,6-(CH_3_)_2_-1,4-benzoquinone	−0.080	6.84 ± 0.42	1.4 ± 0.2 × 10^5^	55.8 ± 3.4	1.0 ± 0.1 × 10^6^
9.	5-OH-1,4-naphthoquinone	−0.090	23.3 ± 1.9	2.8 ± 0.4 × 10^6^	42.2 ± 1.8	7.0 ± 0.6 × 10^6^
10.	5,8-(OH)_2_-1,4-naphthoquinone	−0.110	11.5 ± 1.2	1.8 ± 0.2 × 10^6^	24.7 ± 4.8	5.0 ± 0.5 × 10^7^
11.	1,4-Naphthoquinone	−0.150	7.98 ± 0.40	3.4 ± 0.7 × 10^5^	38.2 ± 2.6	2.4 ± 0.3 × 10^6^
12.	2-CH_3_-1,4-naphthoquinone	−0.200	8.50 ± 0.80	1.6 ± 0.3 × 10^5^	37.9 ± 2.5	1.3 ± 0.2 × 10^6^
13.	(CH_3_)_4_-1,4-benzoquinone (duroquinone)	−0.260	4.71 ± 0.34	8.0 ± 1.0 × 10^4^	115.0 ± 7.0 112.2 ± 4.3 ^a^ 84.3 ± 5.1 ^b^	2.1 ± 0.2 × 10^6^ 1.2 ± 0.1 × 10^6 a^ 1.1 ± 0.1 × 10^6 b^
14.	2-OH-1,4-naphthoquinone	−0.410	0.39 ± 0.05	4.4 ± 0.4 × 10^3^	3.11 ± 0.35	4.0 ± 0.3 × 10^4^
**Single electron acceptors**						
15.	Ferricyanide	0.410	30.4 ± 3.5	1.2 ± 0.2 × 10^5^	102.4 ± 5.9	3.3 ± 0.3 × 10^6^
16.	Cytochrome *c*	0.250	9.66 ± 0.35	1.9 ± 0.2 × 10^5^	104.1 ± 2.2	5.3 ± 0.3 × 10^6^
17.	Fe(EDTA)^−^	0.120	7.30 ± 1.30	7.0 ± 1.0 × 10^3^	11.0 ± 1.2	2.8 ± 0.2 × 10^4^
18.	Benzylviologen	−0.354	6.60 ± 0.40	3.4 ± 0.2 × 10^4^	23.5 ± 1.4	1.7 ± 0.1 × 10^5^
**Nitroaromatic compounds**						
19.	2,4,6-Trinitrophenyl-*N*-methyl- nitramine (tetryl)	−0.191	ND	ND	52.0 ± 2.6	6.8 ± 0.5 × 10^6^
20.	2,4,6-Trinitrotoluene	−0.253	ND	ND	46.4 ± 5.2	1.5 ± 0.1 × 10^5^
21.	*o*-Dinitrobenzene	−0.287	ND	ND	18.9 ± 3.0	8.0 ± 0.7 × 10^4^
22.	*m*-Dinitrobenzene	−0.348	ND	ND	10.1 ± 0.9	8.9 ± 0.6 × 10^3^
23.	*p*-Nitroacetophenone	−0.355	ND	ND	4.67 ± 1.40	2.7 ± 0.3 × 10^4^
24.	5-(1-Aziridinyl)-2,4-dinitro- benzamide (CB-1954)	−0.385	ND	ND	8.83 ± 0.80	2.7 ± 0.2 × 10^4^

^a^—The reaction medium contains H_4_B and Arg; ^b^—The reaction medium contains H_4_B, Arg and 1.0 mM NO_2_-Arg; ND—not determined.

## Data Availability

The original contributions presented in this study are included in this article. Further inquiries can be directed to the corresponding author.

## Appendix A

There exists some uncertainty with the values of *E*^1^_7_ of FMNH/FMNH_2_ couple of nNOS determined in different studies even after their adjustment to pH 7.0 (Table A1).

**Table A1:** Redox potentials of FMNH/FMNH_2_ couple (*E*^1^) of nNOS. Their values, adjusted to pH 7.0 assuming that the reduced FMN is doubly protonated [54], are given in parentheses.

Conditions	*E*^1^ (V)	
	−CAM	+CAM
Flavin reductase domain, pH 7.1 [55]	−0.274 (−0.268)	−0.267 (−0.261)
Holoenzyme, pH 7.0 [54]	−0.220	−0.220 ^a^
Flavin reductase domain, pH 7.5 [56]		−0.300 (−0.270)
Flavin reductase domain, pH 7.4 [41]	−0.199 (−0.175)	−0.284 (−0.260)
Flavin reductase domain, pH 7.6 [39]	−0.276 (−0.240)	

^a^—The absence of effect of CAM has been reported [54].

For this reason, the highest and lowest *E*^1^_7_ values determined in the absence of CAM, −0.175 V and −0.268 V (Table A1), were used in calculations according to Equation (6). Using the data of Figure 1A, we obtain *R*_p_ = 3.9 Å (log *k*_11_ = 3.0), and *R*_p_ = 4.7 Å (log *k*_11_ = 2.0), respectively. Analogously, using *E*^1^_7_ = −0.220 V and *E*^1^_7_ = −0.270 V in the presence of CAM (Table A1) and the data of Figure 1B, we obtain *R_p_* = 2.8 Å (log *k*_11_ = 4.4), and *R*_p_ = 3.1 Å (log *k*_11_ = 4.0), respectively.
